# Supplementary material for: Clustering by phenotype and genome-wide association study in autism
Source: Transl Psychiatry. 2020 Aug 17;10:290. doi: 10.1038/s41398-020-00951-x (PMC7431539; doi:10.1038/s41398-020-00951-x)
Supplement: Supplementary file 2 — Supplementary Fig. 1 [file 41398_2020_951_MOESM2_ESM.pptx]

## Slide 1
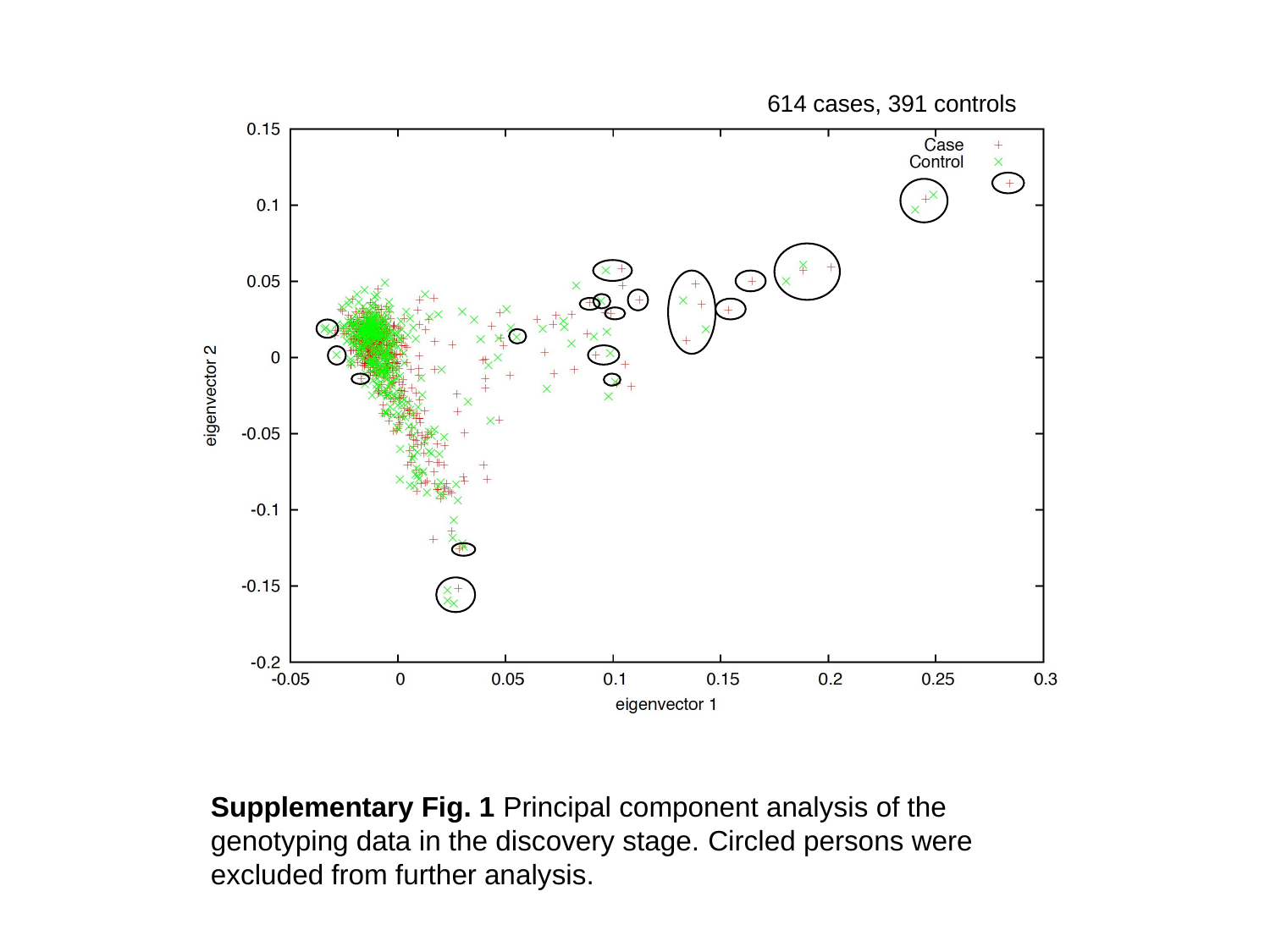

614 cases, 391 controls
Supplementary Fig. 1 Principal component analysis of the genotyping data in the discovery stage. Circled persons were excluded from further analysis.
