# Supplementary figures and images for: Clustering by phenotype and genome-wide association study in autism
Source: Transl Psychiatry. 2020 Aug 17;10:290. doi: 10.1038/s41398-020-00951-x (PMC7431539; doi:10.1038/s41398-020-00951-x)

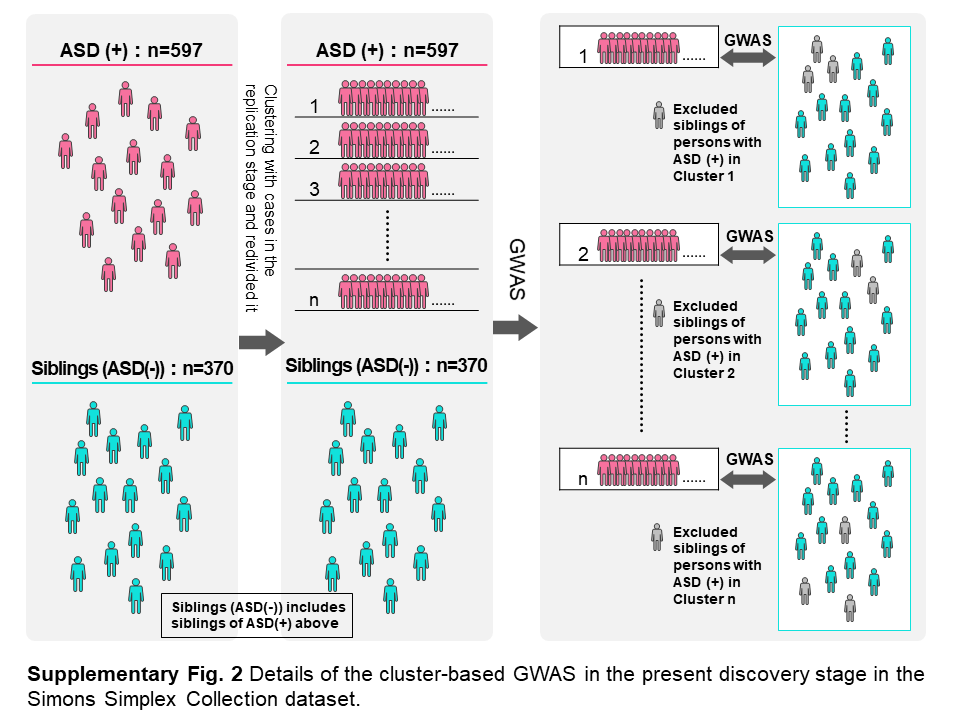

Supplement: Supplementary file 3 — Supplementary Fig. 2 [file 41398_2020_951_MOESM3_ESM.tif]

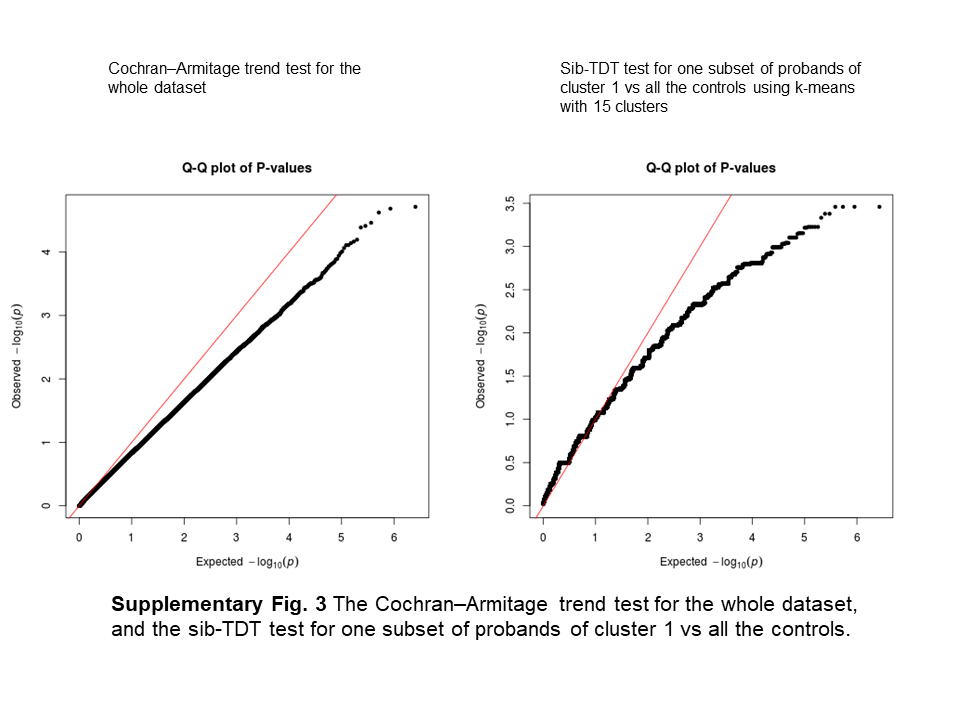

Supplement: Supplementary file 4 — Supplementary Fig. 3 [file 41398_2020_951_MOESM4_ESM.tif]

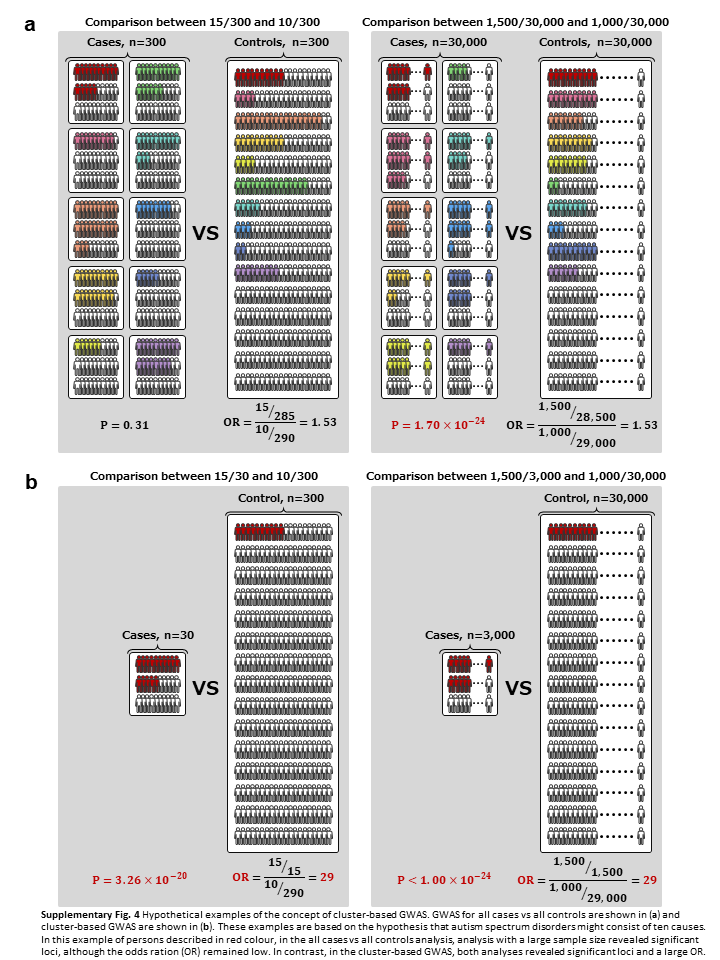

Supplement: Supplementary file 5 — Supplementary Fig. 4 [file 41398_2020_951_MOESM5_ESM.tif]
