# Supplementary material for: Clustering by phenotype and genome-wide association study in autism
Source: Transl Psychiatry. 2020 Aug 17;10:290. doi: 10.1038/s41398-020-00951-x (PMC7431539; doi:10.1038/s41398-020-00951-x)
Supplement: Supplementary file 6 — Supplementary Information 1 [file 41398_2020_951_MOESM6_ESM.docx]

**Supplementary Information 1** Python code of k-means

import pandas as pd

f=pd.read_csv("ssc.csv")

import matplotlib.pyplot as plt

from sklearn.cluster import KMeans

model1=KMeans(n_clusters=5, random_state=0)

model2=KMeans(n_clusters=10, random_state=0)

model3=KMeans(n_clusters=15, random_state=0)

model4=KMeans(n_clusters=20, random_state=0)

data=f[['adi_r_comm_only_verbal_total','adi_r_comm_b_non_verbal_total','adi_r_rrb_c_total','adi_r_soc_a_total','child_vitamins']]

model1.fit(data)

model2.fit(data)

model3.fit(data)

model4.fit(data)

y=model1.labels_

y2=model2.labels_

y3=model3.labels_

y4=model4.labels_

data1=f.copy()

data2=f.copy()

data3=f.copy()

data4=f.copy()

data1['cluster']=y

data2['cluster']=y2

data3['cluster']=y3

data4['cluster']=y4

data1.to_csv("kmeans(c=5).csv")

data2.to_csv("kmeans(c=10).csv")

data3.to_csv("kmeans(c=15).csv")

data4.to_csv("kmeans(c=20).csv")
